# Housing Facts and Fancy

**Published:** 1920-05-08

**Authors:** 


### HOUSING FACTS AND FANCY.

The instances of scandalous housing conditions are interminable, and it must be assumed they will remain so for a while. We gave an instance a week or two ago of how things stood in Southwark. It was stated at a meeting at the Mansion House last week that in St. Pancras there are 11,000 families living in one room; 15,000 families in two rooms; and 10,000 families in three rooms. It is difficult to conceive what this means socially and physically to the families so confined. It is, however, not much use railing at the authorities. That such things should have been permitted before the war is certainly a disgrace, but the accentuation of the evil since the war is the inevitable result of circumstances over which there could have been but little control. However efficient the authorities might have been since the close of hostilities they could not have made up for the lost time, and all that can be done is to urge on, with all possible emphasis, the need for energy and expedition in housing schemes. Some fantastic London Mayor has proposed a house rationing scheme whereby people should be compelled to share habitations with others. It has not even the quality of originality for, like all our extremists, this particular gentleman has borrowed his idea from the Bolshevists, most of whose experiments have proved colossal failures after a period of ruthless application. To take only one particular part of such a plan it would be a jolly task, which might even appal the Bermondsey Council, to distribute the accommodation between the applicants.

To leave foolish irresponsibilities for realities, the papers have recently given particulars of the interesting garden city scheme at Welwyn, about twenty miles from London. Progress has been made with the first fifty houses of this estate, which will ultimately be as big as Bedford. It is an attractive and healthy site of two and a-half thousand acres, and the city will be in full swing in about seven years as an industrial centre much on the same lines of Letchworth. The fifty houses referred to will be ready in six months, and will be occupied by those who are to develop the new city. It will be a valuable part of the decentralisation of London, and a sparkling drop in the ocean of labour needed before all people can be decently, not to say attractively housed.

The best housing scheme for many parts of London and other great cities would be a cleansing fire. Yet a fact, to which *The Times* has directed attention, is that in 1914 a paper was read before the Royal Society of Medicine in which were presented the claims of London as a health resort, and in the discussion which followed the main contention that London is one of the healthiest places in the world was never challenged. With a general mortality of about 14 per 1,000 per annum, with its open spaces covering some 5,000 acres, with its splendid circle of heights, the capital affords many inducements as a place of residence. More than that, London has for long enjoyed a reputation as a good resort for asthmatics. Persons suffering from neurasthenia, too, are said to find benefit often in this stimulating atmosphere. The charm of London remains as potent as ever, the healthiness of London as secure, and we are persuaded (concludes *The Times*) that there is no city in the world, and but few country districts, which can lay claim to greater advantages on the score of health.

